# Supplementary material for: Generating immunogenomic data-guided virtual patients using a QSP model to predict response of advanced NSCLC to PD-L1 inhibition
Source: NPJ Precis Oncol. 2023 Jun 8;7:55. doi: 10.1038/s41698-023-00405-9 (PMC10250344; doi:10.1038/s41698-023-00405-9)
Supplement: Supplementary file 1 — Supplementary Figures and Tables [file 41698_2023_405_MOESM1_ESM.pdf]

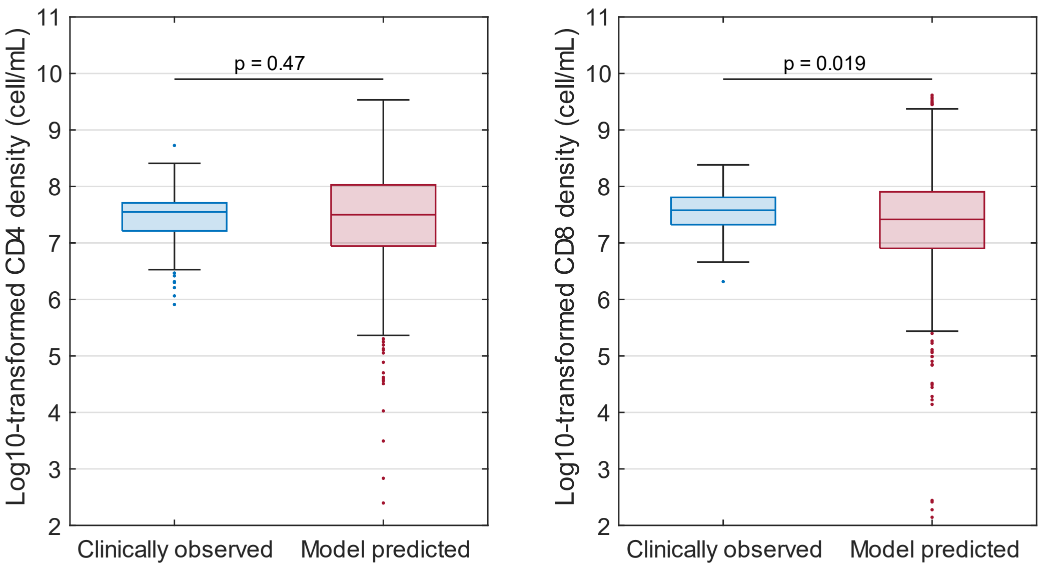

Supplementary Figure 1. Comparison of CD8 and CD4 T cell density distributions between virtual cohort and patient population with stage III NSCLC requested from Kilvaer et al. (PMID: 33035322). 25th, 50th, and 75th percentiles are encoded by box plots with whiskers that define 1.5 times the interquartile range away from the bottom or top of the box. p-values were calculated by Wilcoxon test.

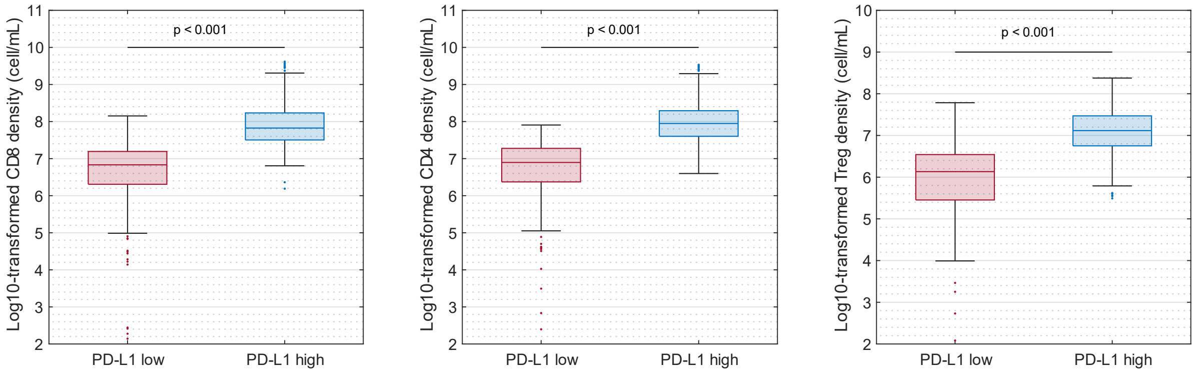

Supplementary Figure 2. Comparison of pre-treatment immune cell densities between PD-L1-low and PD-L1-high groups. 25th, 50th, and 75th percentiles are encoded by box plots with whiskers that define 1.5 times the interquartile range away from the bottom or top of the box. p-values were calculated by Wilcoxon test.

Supplementary Table 1. Comparison between model-predicted pharmacokinetics and clinical observation.

	Cmax,1 (μg/mL)	Cmin,2 (μg/mL)	Cmin,ss (Ctrough,w16; μg/mL)
Model prediction[a]	236 (207, 271; N=629)	66.2 (56.6, 75.9; N=629)	209 (149, 275; N=629)
Clinical measurement (Study 1108)[b]	222 (185, 260; N=838)	81.4 (56.6, 112; N=819)	162 (121, 217; N=334)
Clinical measurement (ATLANTIC trial)[c]	204 (173, 229; N=399)	86.9 (68.9, 107; N=355)	165 (116, 204; N=193)

Median and (25, 75) percentiles are listed with the number of patients/virtual patients. Cmax,1 is the post-first dose maximum durvalumab plasma concentration. Cmin,2 is the pre-second dose minimum concentration. Cmin,ss is the minimum/trough concentrations at steady state (week 16).

[a] US Food and Drug Administration. Clinical pharmacology and biopharmaceutics review(s) for Application number 761069Orig1s000. https://www.accessdata.fda.gov/drugsatfda_docs/nda/2017/761069Orig1s000ClinPharmR.pdf

[b] NCT01693562

[c] NCT02087423

Supplementary Table 2. Model-predicted objective response rate (ORR) and immune subset ratios in virtual patients selected by various data combinations.

	Predicted ORR (95% CI)	CD8/Treg (95% CI)	CD8/CD4 (95% CI)	M1/M2 (95% CI)
Immunogenomic data[a]	N/A	4.9 (0.9, 121.9)	0.9 (0.2, 10.9)	0.22 (0.01, 0.90)
Plausible patients	16.8 (11.3, 22.3)%	5.1 (0.6, 87.7)	1.1 (0.2, 8.8)	0.31 (0.03, 5.13)
VP filtered by M1/M2	16.6 (11.3, 22.3)%	5.4 (0.6, 150.6)	0.8 (0.1, 6.2)	0.24 (0.02, 2.34)
VP filtered by CD8/Treg	16.1 (10.6, 21.9)%	5.0 (0.7,114.1)	0.8 (0.1, 5.1)	0.22 (0.02, 1.88)
VP filtered by CD8/CD4	18.6 (13.3, 24.2)%	5.3 (0.7, 112.6)	0.8 (0.1, 5.7)	0.24 (0.02, 2.37)
VP filtered by M1/M2 and CD8/Treg	15.9 (10.9, 21.1)%	4.9 (0.6, 108.5)	0.8 (0.1, 7.3)	0.22 (0.02, 2.25)
VP filtered by M1/M2 and CD8/CD4	18.8 (13.7, 24.6)%	5.4 (0.6, 99.3)	0.8 (0.1, 6.1)	0.24 (0.02, 1.97)
VP filtered by CD8/Treg and CD8/CD4	17.80 (12.70, 23.83)%	5.3 (0.7, 140.7)	0.8 (0.1, 6.3)	0.24 (0.02, 3.23)
VP filtered by LUSC	20.6 (14.5, 27.0)%	7.0 (0.8, 159.6)	1.1 (0.1, 9.8)	0.26 (0.02, 2.73)
VP filtered by LUAD	16.5 (11.1, 22.7)%	4.0 (0.7, 79.9)	0.7 (0.1, 4.9)	0.20 (0.02, 3.23)
Virtual patients	18.6 (13.3, 24.2)%	5.0 (0.5, 178.3)	0.8 (0.1, 6.5)	0.22 (0.02, 2.53)

[a] Data points that contained zero value(s) for immune cell proportions were removed to avoid singularities.

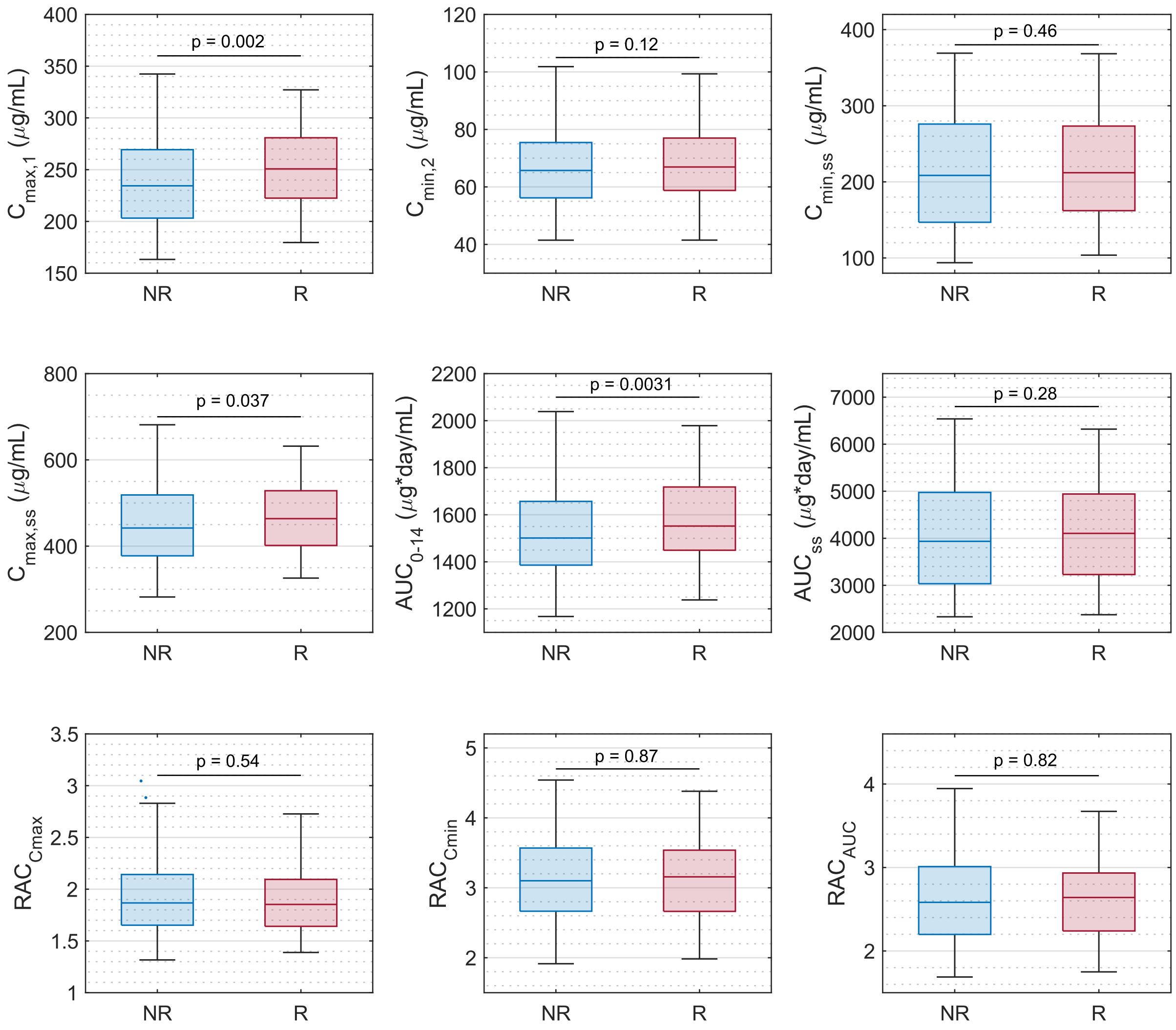

Supplementary Figure 3. Comparison of pharmacokinetic variable distributions between responders (R) and non-responders (NR). Cmax,1 is the post-first dose maximum durvalumab plasma concentration. Cmin,2 is the pre-second dose minimum concentration. Cmax,ss and Cmin,ss are the maximum and minimum concentrations at steady state (week 16), respectively. AUC0-14 is the area under the concentration curve from day 0-14. RACs are the drug accumulation ratios of Cmax,ss/Cmax,1, Cmin,ss/Cmin,2, and AUCss/AUC0-14. 25th, 50th, and 75th percentiles are encoded by box plots with whiskers that define 1.5 times the interquartile range away from the bottom or top of the box. p-values were calculated by Wilcoxon test.

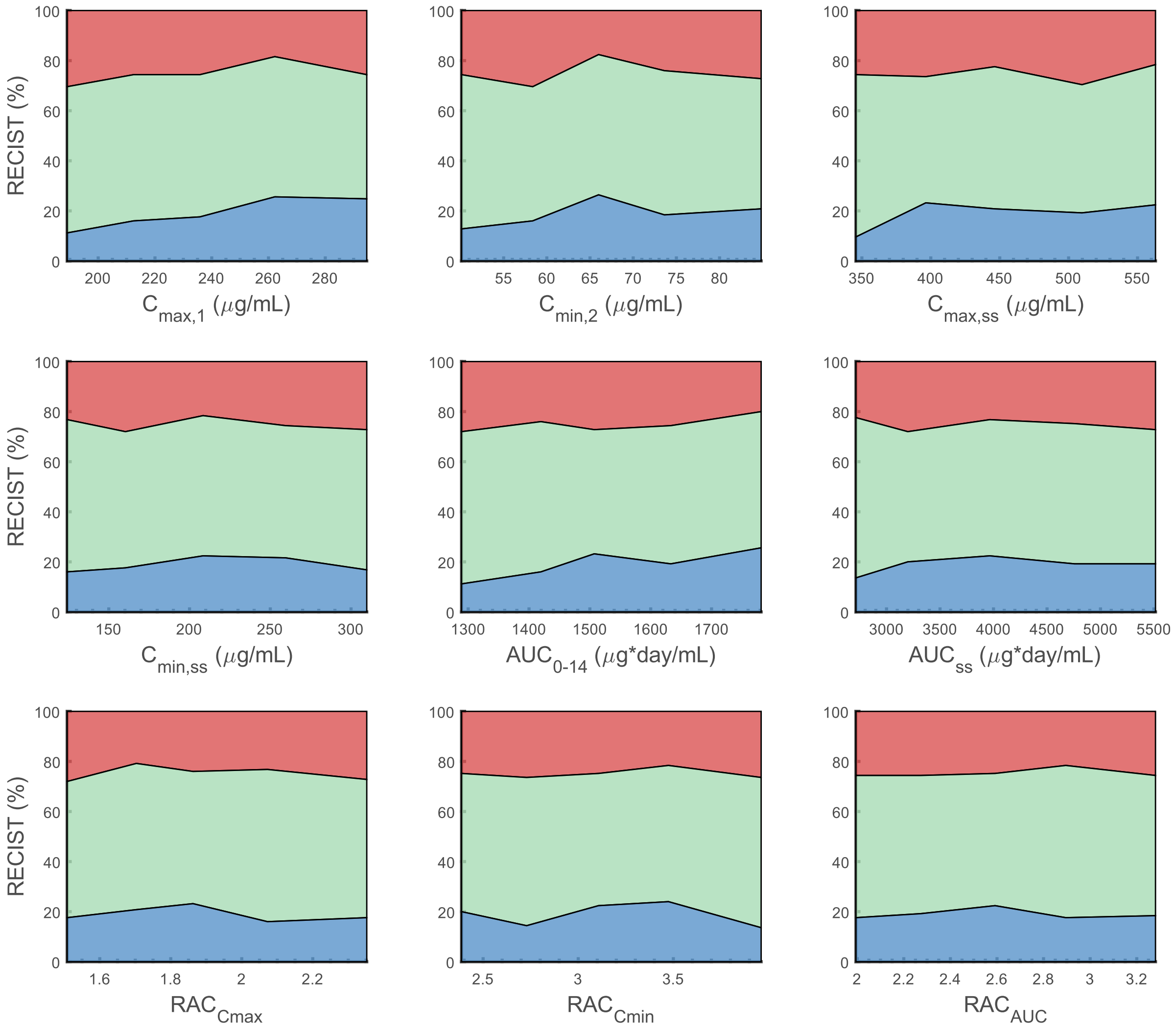

Supplementary Figure 4. Effect of pharmacokinetic variables on objective response. For each variable of interest, virtual patients are sorted by the variable amount in ascending order, and evenly divided into 5 subgroups. The response status of each subgroup is plotted against the corresponding median variable amount. Blue represents partial or complete response. Green represents stable disease. Red represents progressive disease.

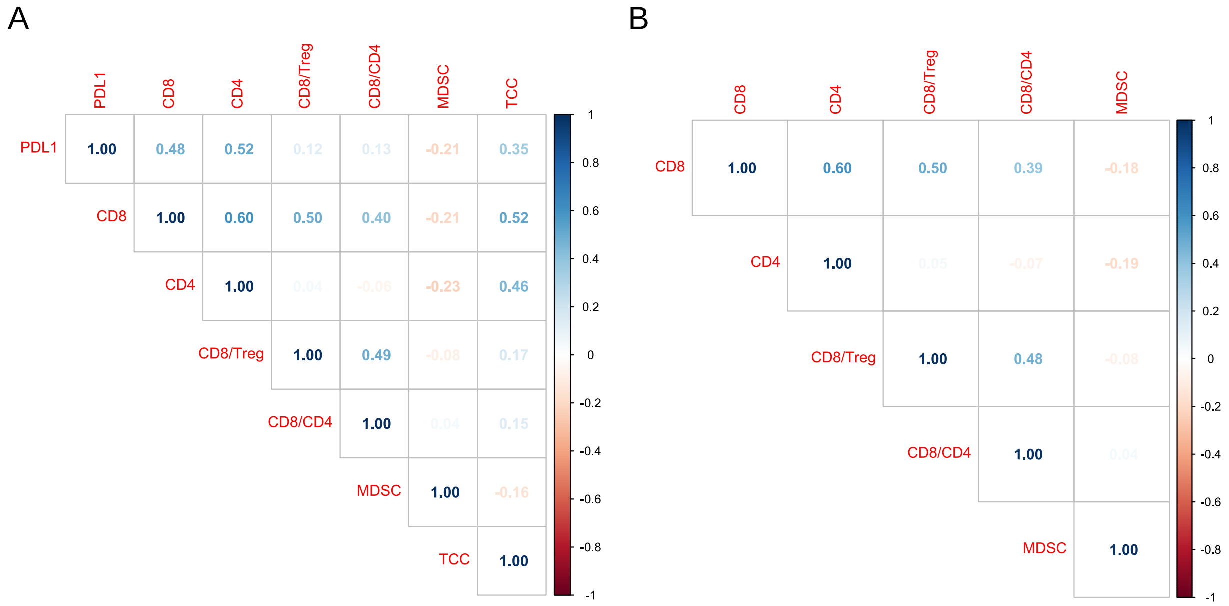

Supplementary Figure 5. Correlation matrix of (A) pre-treatment and (B) on-treatment variables. Treg, regulatory T cell. MDSC, myeloid-derived suppressor cells. TCC, the number of NSCLC-specific T cell clones.

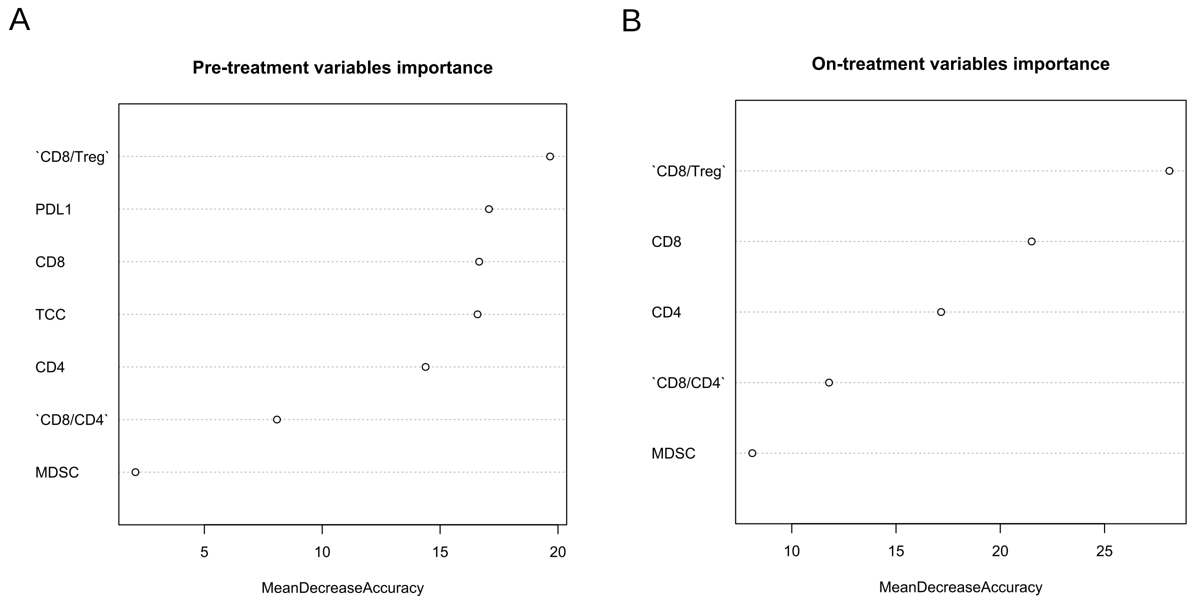

Supplementary Figure 6. Variable importance calculated by random forest models of (A) pre-treatment and (B) on-treatment biomarkers of interest. Treg, regulatory T cell. MDSC, myeloid-derived suppressor cells. TCC, the number of NSCLC-specific T cell clones.

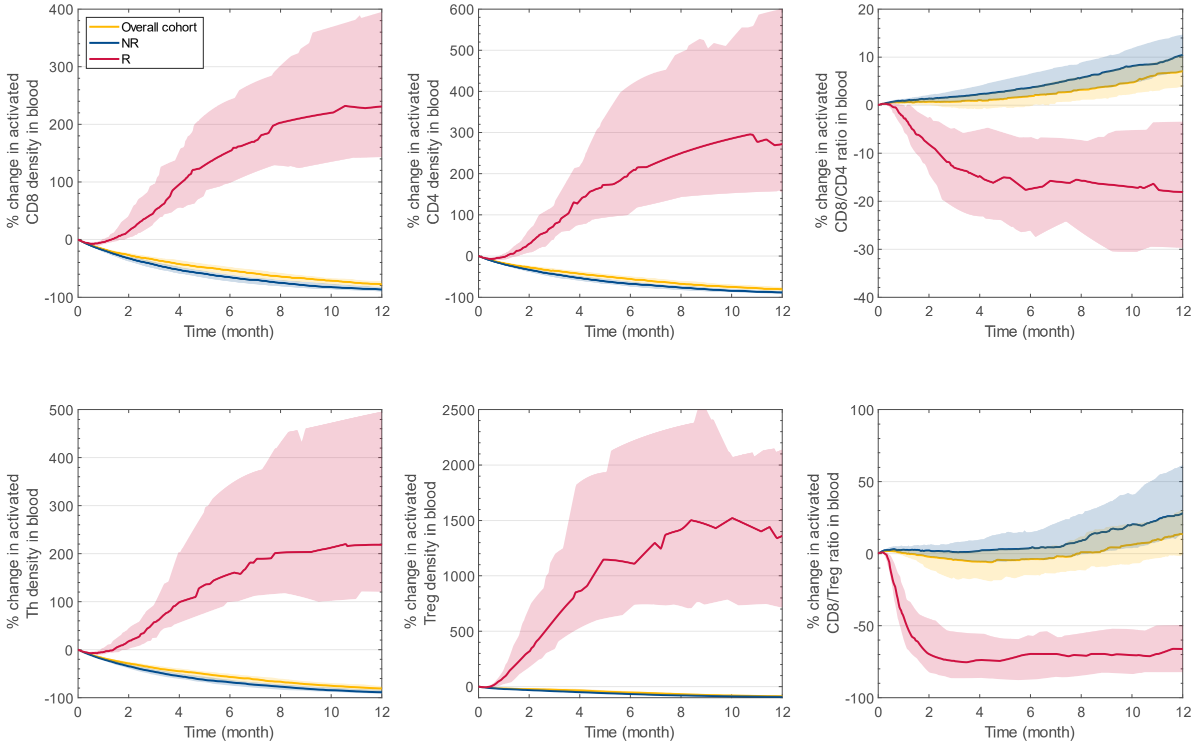

Supplementary Figure 7. Effect of PD-L1 inhibition on model variables in the central compartment in responders, non-responders, and the overall virtual patient cohort, including percentage change in activated CD8 T cell, activated CD4 T cell, CD8/CD4 ratio, helper T cell (Th), regulatory T cell (Treg), and CD8/Treg ratio. Solid lines represent median values. Shaded areas represent 95 percentile bootstrap confidence intervals.

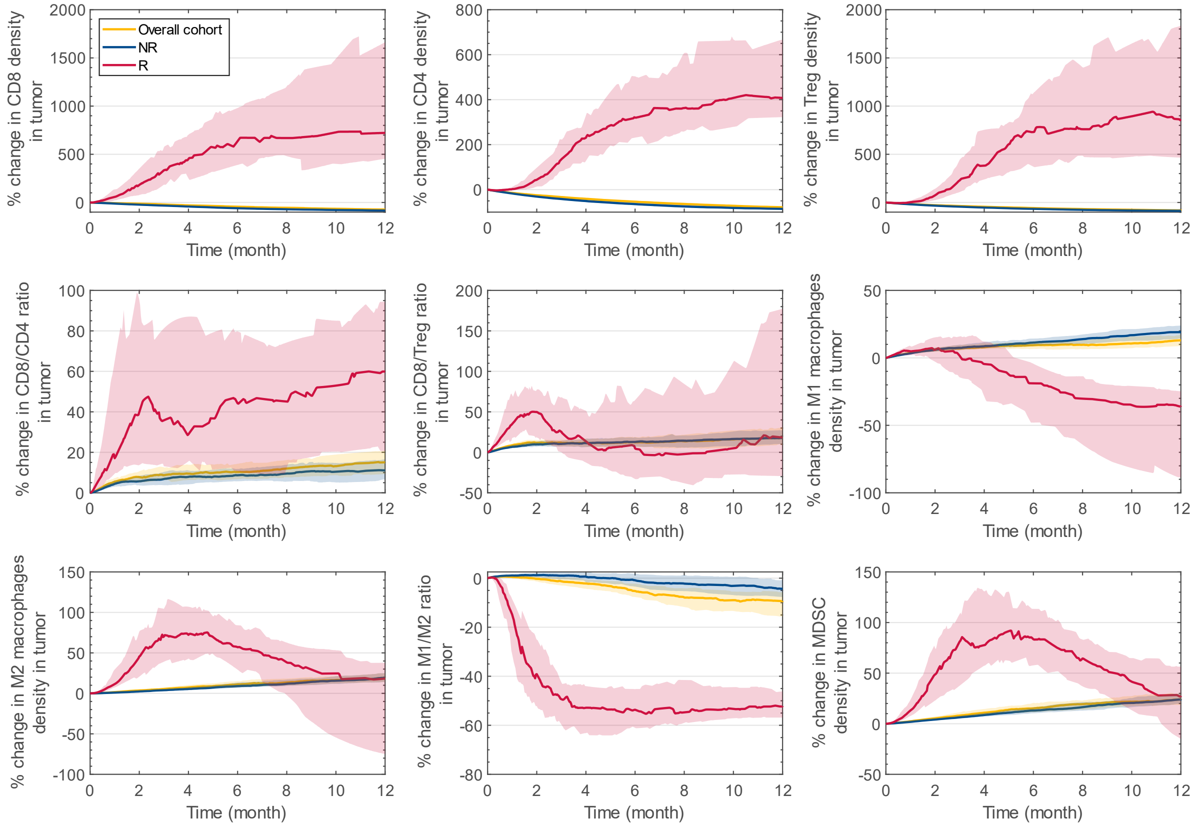

Supplementary Figure 8. Effect of PD-L1 inhibition on model variables in the tumor compartment in responders (R), non-responders (NR), and all the virtual patients: percentage change in CD8 T cell, CD4 T cell, regulatory T cell (Treg), CD8/CD4 ratio, CD8/Treg ratio, M1 macrophages, M2 macrophages, M1/M2 ratio, and myeloid-derived suppressor cell (MDSC). Solid lines represent median values. Shaded areas represent 95 percentile bootstrap confidence intervals.

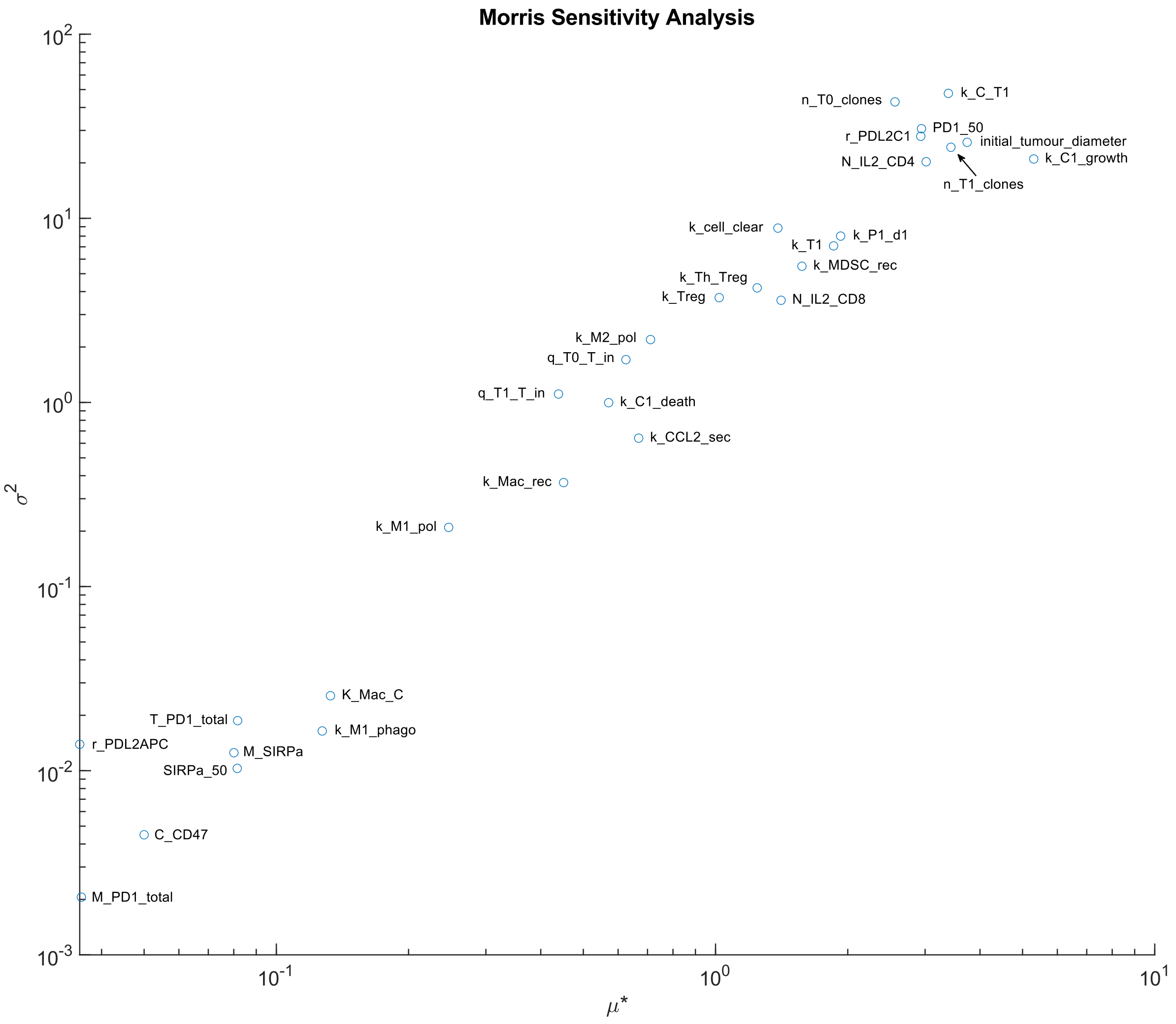

Supplementary Figure 9. Global sensitivity analysis using Morris screening method. Ranking of parameters determining tumor size at the end of durvalumab treatment (day 400). μ^*, estimate of mean absolute value of elementary effects; σ^2, estimate of variance of elementary effects.
